# Discriminant Validity of the 20-Item Toronto Alexithymia Scale and the Perth Alexithymia Questionnaire in Relation to Psychological Distress

**DOI:** 10.3390/bs16030339

**Published:** 2026-02-28

**Authors:** R. Michael Bagby, Ardeshir Mortezaei, Sharlane C. L. Lau, Cheyenne S. McIntyre, Graeme J. Taylor

**Affiliations:** 1 Department of Psychology, University of Toronto, Toronto, ON M1C 1A4, Canada; 2 Department of Psychiatry, University of Toronto, Toronto, ON M1C 1A4, Canada; graeme.taylor@utoronto.ca; 3 Department of Psychology, D’Youville University, Buffalo, NY 14201, USA; ardeshir.mortezaei@ontariotechu.net; 4 Graduate Center, City University of New York, New York, NY 10016, USA; clau@gradcenter.cuny.edu; 5 Department of Psychological Science, Ball State University, Muncie, IN 47306, USA; cheyenne.mcintyre@bsu.edu

**Keywords:** alexithymia, Toronto Alexithymia Scale, Perth Alexithymia Questionnaire, psychological distress, discriminant validity

## Abstract

Recent investigations have questioned whether the Toronto Alexithymia Scale (TAS-20) distinguishes alexithymia from general psychological distress, with some researchers positioning the Perth Alexithymia Questionnaire (PAQ) as demonstrating superior discriminant validity. We evaluated both instruments in a community sample enriched with participants having psychiatric diagnoses or a treatment history (*N* = 681). Participants completed the TAS-20, PAQ, and Depression Anxiety Stress Scales-21 (DASS-21). Both alexithymia measures showed moderate correlations with the DASS-21, suggesting related but distinct constructs. Item-level exploratory factor analysis revealed a factor separation between general emotional dysfunction and distress, with no meaningful cross-loadings for TAS-20 Difficulty Identifying Feelings (DIF) items. At the subscale level, a two-factor solution supported distinct general emotional dysfunction and distress factors. In contrast to two recent investigations, the TAS-20 DIF subscale loaded exclusively on a general emotional dysfunction factor with negligible loading on a distress factor. These findings demonstrate that neither the TAS-20 nor the PAQ is compromised by distress, challenging recent claims of TAS-20 discriminant validity problems and underscoring the importance of replication across diverse samples and analytic methods.

## 1. Introduction

Alexithymia, as originally conceptualized through clinical observations (Nemiah et al., 1976; Taylor et al., 1997), refers to a stable personality trait characterized by difficulties identifying and describing emotional feelings, an externally oriented or stimulus-bound thinking style, and a limited capacity for imagination and fantasy. This diminished ability to identify, communicate, and process affect is considered a risk factor for a range of medical and psychiatric disorders (Bagby et al., 2020; Luminet et al., 2018; Taylor et al., 1997, 2024). The 20-item Toronto Alexithymia Scale (TAS-20; Bagby et al., 1994a, 1994b) was designed to assess alexithymia according to this original conceptualization and comprises three highly replicable factor subscales: Difficulty Identifying Feelings (DIF), Difficulty Describing Feelings (DDF), and Externally Oriented Thinking (EOT) (Schroeders et al., 2022).^1^

Recent investigations have questioned whether the TAS-20 adequately distinguishes alexithymia from psychological distress (see e.g., Preece et al., 2020, 2024), prompting a resurgence of discussion and empirical evaluation (Bagby et al., 2020; Taylor et al., 2024). Over the years, two methodologies have primarily been used to address this issue: (1) longitudinal investigations examining the absolute and relative stability of TAS-20 scores, and (2) factor analytic studies in which the items or subscales of the TAS-20 and various measures of psychological distress are jointly factor analyzed. Although both methods are useful in disentangling the relationship between psychological distress and alexithymia, most of the recent concerns about the discriminant validity of the TAS-20 have been driven by the second methodological approach.

### 1.1. Stability and Change in TAS-20 Scores in Patient Samples

A critical issue in evaluating whether alexithymia reflects a stable personality trait, or a transient expression of psychological distress involves distinguishing *absolute* from *relative* stability. Absolute stability refers to *mean-level consistency* over time; that is, whether the average TAS-20 scores within a group increase or decrease as clinical symptoms change. Relative stability, by contrast, reflects *rank-order consistency*, or the extent to which individuals maintain their relative standing on alexithymia compared to others despite fluctuations in mood or distress. Longitudinal designs, particularly in clinical populations undergoing treatment and assessed pre- and post-intervention, provide an ideal framework for evaluating both forms of stability and directly testing whether the TAS-20 primarily indexes enduring personality features or transient affective states.

Empirical evidence addressing this distinction is provided by longitudinal studies that track alexithymia across phases of illness and treatment, allowing direct evaluation of both absolute and relative stability. Across diverse patient groups assessed at least twice during illness or treatment, including major depressive disorder (Luminet et al., 2001; Saarijärvi et al., 2006), functional gastrointestinal disorders (Porcelli et al., 2003), alcohol dependence (de Timary et al., 2008; Thorberg et al., 2016), obsessive–compulsive disorder (Rufer et al., 2004), and breast cancer (Luminet et al., 2007), TAS-20 scores typically show modest mean-level change, suggesting limited absolute stability and some state-sensitivity, especially in the DIF and DDF facets. However, relative stability, indexed by test–retest correlations, is consistently moderate-to-large across intervals ranging from several weeks to multiple years, even when depressive or anxiety symptoms change substantially. These findings demonstrate that although the TAS-20 is not entirely invariant to distress levels, it nonetheless captures a stable, trait-like core of alexithymia that persists despite symptomatic fluctuations. This pattern of moderate-to-high relative stability occurring alongside limited absolute stability in the context of symptom reduction is a well-established characteristic of psychological vulnerability factors (Kotov et al., 2010a, 2010b; Ormel et al., 2013).

### 1.2. First-Order Factor Analytic Studies

Five first-order, item-level factor analytic studies have jointly examined TAS-20 items along with items from well-validated distress measures, including the Beck Depression Inventory (BDI), Hospital Anxiety and Depression Scale (HADS), Self-Rating Depression Scale (SDS), and Screening List for Somatization Symptoms (SOMS). These investigations varied in sample type and factor analytic approach. The results from these studies are summarized in Table 1.

As displayed in Table 1, across eight independent samples totaling 3561 participants from patient, university, and community settings, using varied distress measures and factor analytic approaches, a consistent pattern emerged. TAS-20 items (or items from the 26-item TAS) loaded significantly on alexithymia factors (DIF, DDF, EOT), clearly separable from distress items, which typically formed separate depression, anxiety, and somatization factors. Despite variation in the number of retained factors (4–6) and variance explained (27.0% to 48.6%), the structural separation of alexithymia and distress replicated consistently across samples and measures. While minor exceptions to this pattern emerged, they were limited and did not meaningfully challenge the overall distinctiveness of the domains. Parker et al. (1991) and Hintikka et al. (2001), for example, each recovered three alexithymia factors distinct from depression factors; Müller et al. (2003) found TAS-20 and SDS (i.e., depression content items) loaded on separate factors. Bach et al. (1996) demonstrated separable TAS-20 and SOMS (i.e., somatic complaint content items) factors in both clinical and non-clinical samples, ruling out overlap with somatization. And Marchesi et al. (2000) reported distinct TAS-20 factors with only minor DIF–anxiety cross-loadings in a mixed psychiatric and non-psychiatric patient sample.

### 1.3. First-Order Versus Second-Order Factor Analyses

Preece et al. (2020, 2024) argue that first-order, item-level factor analysis does not offer a strong test of discriminant validity, noting that general distress often manifests as a second-order factor above first-order depression, anxiety, and stress dimensions. They further reason that because alexithymia is typically operationalized and interpreted via its subscales, discriminant validity is best examined at the subscale level through second-order factor analysis. An alternative view rooted in classical test theory emphasizes that the most direct evidence for discriminant validity lies in relationships among manifest indicators, or the items themselves, which represent the core content of constructs (Cronbach & Meehl, 1955). When first-order analyses consistently show that TAS-20 items do not cross-load with distress items, this provides strong evidence of empirical distinctiveness at the construct’s core.

By contrast, second-order analyses aggregate items into subscales, introducing shared method variance (similar wording, scale format, or response tendencies) that can artificially inflate subscale correlations and create the appearance of construct overlap even when underlying item content is in fact distinct. Second-order results should therefore complement, rather than replace, item-level evidence. When item-level analyses consistently demonstrate that TAS-20 items form distinct factors from distress items, this represents particularly compelling evidence for discriminant validity, as it directly addresses whether the manifest content of the measures overlaps.

### 1.4. Second-Order Factor Analytic Studies

Table 2 summarizes investigations examining the relationship between alexithymia and psychological distress at the subscale or second-order level. As with first-order investigations, these studies varied in sample composition, measures, and analytic methods. Alexithymia measures included the Toronto Alexithymia Scale-Revised (TAS-R; Taylor et al., 1992), TAS-20 (Bagby et al., 1994a, 1994b), Bermond-Vorst Alexithymia Questionnaire (BVAQ; Vorst & Bermond, 2001), and Perth Alexithymia Questionnaire (PAQ; Preece et al., 2018). Distress measures included the Depression Anxiety Stress Scales-21 (DASS-21; Lovibond & Lovibond, 1995), Symptom Checklist-90-Revised (SCL-90-R; Derogatis, 1983), Inventory of Interpersonal Problems-64 (IIP-64; Horowitz et al., 2000), and Mississippi Scale for Combat-Related PTSD (MPTSD; Keane et al., 1988).

Early studies using principal components analysis with varimax rotation suggested limited discriminant validity. Badura (2003) reported that TAS-R and MPTSD subscales loaded on a single factor among male combat veterans, which was interpreted as a substantial overlap of alexithymia and post-traumatic distress. Similarly, Leising et al. (2009) reported one broad factor combining the TAS-20, SCL-90-R, and IIP-64 subscales (~60% variance), indicating a general distress dimension rather than distinct constructs, though this study had a small sample size (*N* = 63).

More recent work using principal axis factoring with oblimin rotation evaluated distinct higher-order dimensions. Preece et al. (2020) conducted second-order analyses of the TAS-20, BVAQ, and PAQ subscales with DASS-21 subscales across five Australian samples (three completed the TAS-20). They consistently found two correlated factors with one representing alexithymia, the other distress, but noted that the TAS-20 DIF subscale cross-loaded on the distress factor, whereas the BVAQ and PAQ subscales did not. Preece et al. (2024) subsequently replicated this pattern in a U.S. community sample (*N* = 508), again reporting TAS-20 DIF cross-loading but clean PAQ separation. On this basis, they concluded that the TAS-20 is partially confounded with distress.

It is important to note, however, that in a follow-up first-order analysis using only the seven TAS-20 DIF items and all DASS-21 items, Preece et al. (2020) found that none of the DIF items cross-loaded on the depression, anxiety, or stress factors formed by DASS-21 items, supporting the discriminant validity of the DIF facet at the item level.

As summarized in Table 2, these second-order studies yield a different pattern than the item-level findings. Whereas the first-order analyses consistently show that TAS-20 items form distinct alexithymia factors, the second-order analyses suggest partial overlap, particularly for DIF, when the data are aggregated at the subscale level. This suggests that the apparent construct overlap found in some second-order analyses may reflect the influence of scale aggregation rather than a genuine construct confounding of item content. This interpretation is strongly supported by the robust item-level evidence of distinctiveness found here and in previous studies.

### 1.5. The Present Study

The present study evaluated the discriminant validity of the TAS-20 and PAQ in relation to psychological distress, extending previous investigations in two ways. First, we conducted item- and subscale-level analyses concurrently within the same sample, allowing us to determine whether apparent overlap with distress is driven by specific TAS-20 DIF items, as suggested in prior work (see Preece et al., 2020, 2024), or by the analytic method itself (item-level vs. subscale-level). This dual approach provides a more rigorous and comprehensive test of discriminant validity than either method alone. Second, we used a community sample enriched with participants having a history of psychiatric diagnosis and treatment, thereby increasing variability in both alexithymia and distress and providing a more stringent test of discriminant validity.

## 2. Materials and Method

### 2.1. Sample and Procedure

The sample comprised 681 community adults from the U.S. recruited through the online platform Prolific. Participants were paid $7.00 and were required to currently reside in the U.S. and read English fluently. After providing informed consent, participants completed a battery of questionnaires, including the TAS-20, PAQ, and DASS-21, via the online platform. Data were screened for invalid responding using Prolific criteria, yielding a final sample of 681 participants (329 men, 351 women, one participant chose not to identify gender). A subset of these data was used in a previous study (Bagby et al., 2025) that addressed different research questions. The study was conducted in accordance with the Declaration of Helsinki, and the protocol was approved by the Research Ethics Board of the University of Toronto (Protocol No. 00046044) on 7 March 2024.

The mean age was 46.23 years (SD = 16.68), and the mean years of education were 15.70 (SD = 2.80). Self-identified ethno-racial backgrounds were: 63.2% White, 13.9% Black, 8.3% Mixed/Multi-racial/Bi-racial, 6.8% Central American/Hispanic/Latinx, 2.7% East Asian, 1.5% Southeast Asian, 1.2% South Asian, 0.8% Indigenous/First Nations/Aboriginal/Native American, 0.5% Middle Eastern, 0.2% Middle or Central Asian, and 0.8% preferred not to respond. Of the 681 participants, 189 (27.75%) reported receiving a psychiatric diagnosis (past or current), and 272 (39.9%) reported receiving treatment for mental health issues at some point, with 142 (20.85%) currently in treatment and 130 (19.1%) previously treated.

The sample size was also well-suited for the planned factor-analytic procedures. Contemporary methodological work indicates that sample size requirements in factor analysis depend more on communalities and the degree of factor overdetermination (i.e., number of indicators per factor) than on fixed numerical rules of thumb (MacCallum et al., 2001). Simulation studies demonstrate that when factors are well-defined and items show adequate communalities, stable factor recovery can be achieved with substantially smaller samples, and precision improves further as N increases (Mundfrom et al., 2005; de Winter et al., 2009). Given the large number of indicators across instruments and the present sample size (*N* = 681), the study was well-powered to yield stable and interpretable factor solutions.

### 2.2. Measures

#### 2.2.1. Toronto Alexithymia Scale-20 (TAS-20)

The TAS-20 (Bagby et al., 1994a, 1994b) is a 20-item self-report questionnaire designed to assess alexithymia according to the original, affect deficit conceptualization of the construct (Sifneos, 1987, 1994; Taylor et al., 1997). Items are rated on a five-point Likert scale, with higher scores reflecting greater alexithymia severity. Although the authors recommend using only the total score, the instrument comprises three subscales—Difficulty Identifying Feelings (DIF; 7 items), Difficulty Describing Feelings (DDF; 5 items), and Externally Oriented Thinking (EOT; 8 items). The TAS-20 has demonstrated good validity and reliability (Bagby et al., 2020; Sekely et al., 2018). While the EOT subscale sometimes shows lower internal consistency than the DIF and DDF subscales, coefficient alphas and omegas across studies are adequate for a subscale that is multidimensional in content (Clark & Watson, 2019). The factor structure is stable and highly replicable (Schroeders et al., 2022).

#### 2.2.2. Perth Alexithymia Questionnaire (PAQ)

The PAQ (Preece et al., 2018) is a 24-item self-report measure of alexithymia grounded in the attention-appraisal model of the construct (Preece et al., 2017). It assesses three core features—Difficulty Identifying Feelings (DIF), Difficulty Describing Feelings (DDF), and Externally Oriented Thinking (EOT)—with valence-specific subscales distinguishing between negative and positive emotions for DIF and DDF. Items are rated on a seven-point Likert scale. The instrument yields five subscales: Negative-DIF (N-DIF), Negative-DDF (N-DDF), Positive-DIF (P-DIF), and Positive-DDF (P-DDF), each comprising four items, and General-EOT (G-EOT; 8 items). A total score is calculated by summing all 24 items. Higher scores indicate greater difficulties and more severe alexithymia. The PAQ has demonstrated good psychometric properties (Preece et al., 2018). Although the factor structure has been replicated in student, community, and clinical samples, some evidence suggests excessive item redundancy and high inter-item correlations within and between subscales, raising concerns that the factor structure may be over-fitted (Bagby et al., 2025; Zahid et al., 2023).

#### 2.2.3. Depression Anxiety Stress Scales-21 (DASS-21)

The DASS-21 (Lovibond & Lovibond, 1995) is a 21-item self-report instrument measuring symptoms of depression (DEP), anxiety (ANX), and stress (STR), with seven items per subscale. Items are rated on a four-point Likert scale reflecting experiences over the past week, with higher scores indicating greater symptom severity. Separate subscale scores and a total score representing general psychological distress can be calculated. The DASS-21 demonstrates good to excellent internal consistency across community and clinical samples (Henry & Crawford, 2005) and strong convergent and discriminant validity with related measures of affective symptoms (e.g., BDI-II, HADS). Confirmatory factor analyses typically support the intended three-factor structure, with some studies also identifying a higher-order general distress factor (Lee et al., 2019; Sinclair et al., 2012). It is one of the most widely used screening measures of psychological distress in research and clinical contexts (Antony et al., 1998).

## 3. Results

### 3.1. Descriptive Statistics and Total Scale and Subscale Internal Reliabilities

Descriptive statistics for the TAS-20, PAQ, and DASS-21 total and subscale scores are presented in Table 3, including means, standard deviations, scores ranges, and distribution indices (skewness and kurtosis). All variables were approximately normally distributed, with skewness and kurtosis values well within commonly accepted thresholds (absolute skew < 2.0 and absolute kurtosis < 7.0; West et al., 1995).

Internal consistency was evaluated using three complementary indices: Cronbach’s coefficient α (Cronbach, 1951), McDonald’s coefficient ω (McDonald, 1999), and the average inter-item correlation (AIC; Clark & Watson, 1995). When evaluating these estimates, construct breadth and scale length must be considered. For narrow, homogeneous constructs, Clark and Watson (2019) recommend α and ω values between 0.75 and 0.90, with AIC values between 0.30 and 0.50; values exceeding these upper bounds indicate excessive redundancy. For broader, heterogeneous constructs encompassing multiple facets, somewhat lower estimates are acceptable (α and ω: 0.70–0.85; AIC: 0.25–0.40). Additionally, because α and ω increase with scale length even when average inter-item correlations remain constant, values approaching or exceeding 0.95 on longer scales (≥20 items) warrant scrutiny as potential indicators of item redundancy.

Table 4 displays internal reliability estimates for all total and subscale scores. Most estimates fell within or near the acceptable ranges, although the PAQ total and subscale scores all exceeded recommended parameters, suggesting excessive item redundancy. The TAS-20 EOT subscale estimates were smaller than generally recommended but marginally acceptable given the heterogeneity of its item content.

### 3.2. Correlations Among the TAS-20, PAQ, and DASS-21

Correlations among the TAS-20, PAQ, and DASS-21 total and subscale scores are shown in Table 5. We used Cohen’s (1988) guidelines to classify correlations as small (0.10), medium (0.30), large (0.50), or very large (0.80). Meta-analytic investigations have shown that associations between personality traits serving as vulnerability factors for mental disorders and accompanying symptoms range between *r* = 0.25 and *r* = 0.60, with correlations from 0.30 to 0.50 considered “typically meaningful” (Clark & Watson, 1995, 2019; Kotov et al., 2010a, 2010b), while those exceeding *r* = 0.70 represent cross-construct redundancy.

As displayed in Table 5, almost all correlations between the TAS-20 and PAQ scores and the DASS-21 scores were large, with none exceeding r = 0.70 and most falling within the typically meaningful range. The exceptions were the EOT subscales for both instruments, which in some cases fell below *r* = 0.25. Overall, the TAS-20 and PAQ showed comparable associations with general psychological distress and its components (depression, anxiety, and stress) as measured by the DASS-21. The DIF and DDF subscales demonstrated associations commonly observed in personality-psychopathology vulnerability models. This pattern is compatible with related but distinct constructs.

### 3.3. Factor Analyses

#### 3.3.1. Item-Level, First-Order Factor Analysis

To examine the structural distinctiveness of alexithymia and psychological distress, following procedures from earlier item-level factor analyses, the 65-item correlation matrix (20 TAS-20 items, 24 PAQ items, 21 DASS-21 items) was subjected to exploratory factor analysis (EFA). Prior to EFA, the distributional properties of all items were examined to ensure they approximated a normal distribution. Items from all three instruments showed neither excessive skew nor kurtosis, supporting the use of the correlation matrix for factoring. The Kaiser–Meyer–Olkin measure of sampling adequacy (0.97) and Bartlett’s test of sphericity (χ^2^(2080) = 31,427.64, *p* < 0.001) indicated the data were suitable for factor analysis (Bartlett, 1954; Hutcheson & Sofroniou, 1999; Kaiser, 1974). Inspection of communalities revealed that all items exceeded the recommended minimum (0.30), supporting factorability. The correlation matrix determinant was greater than 0.001, indicating no multicollinearity issues.

We used principal axis factoring (PAF) extraction with oblique (oblimin) rotation (Δ = 0) to allow for correlated factors. To determine the optimal number of factors, we performed Horn’s (1965) parallel analysis (PA) and Velicer’s (1976) minimum average partial (MAP) test, supplemented by scree plot inspection. Both PA and MAP indicated eight factors, and the scree plot showed a break following the eighth factor. We rotated eight factors to simple structure, accounting for 64.16% of the total variance. Table 6 displays the factor loading matrix, eigenvalues, percentage of variance explained by each factor, and cumulative variance. Consistent with conventional guidelines (Stevens, 2002; Tabachnick & Fidell, 2019), factor loadings ≥0.40 were interpreted as meaningful; ≥0.30 and <0.40 as marginally meaningful; ≥0.20 and <0.30 as non-meaningful; and <0.20 as trivial.

Although PA, MAP, and eigenvalue criteria suggested eight factors, only the first three accounted for meaningful variance using the conventional 5% threshold (Stevens, 2002). Factors 1, 2, and 3 explained 37.99%, 10.22%, and 4.57% of variance, respectively, together accounting for more than 50% of the total variance. In contrast, Factors 4 through 8 each explained less than 3% of variance (range: 1.92% to 2.78%), falling below conventional thresholds for interpretation (Stevens, 2002). Most of these factors had no salient loadings. Specifically, Factor 4 did not include any loadings ≥0.40; the highest observed loadings were small (e.g., DASS Q15, λ = 0.234; DASS Q16, λ = −0.274; DASS Q17, λ = −0.257), with no secondary loadings approaching conventional interpretive thresholds. Factor 5 similarly showed no meaningful loadings, with the largest coefficients in the marginal or non-meaningful range (e.g., TAS-20 Q2, λ = 0.211). Factor 6 was characterized by diffuse and small coefficients (e.g., TAS-20 Q3, λ = 0.240), with all remaining loadings < 0.30. Factor 7 was anchored by a single meaningful loading from TAS-20 Q5 (EOT; λ = 0.434), but no additional items loaded ≥0.30 on this factor. Factor 8 did not contain any meaningful loadings, with coefficients generally falling within trivial to non-meaningful ranges. In each case, the absence of multiple convergent indicators and the predominance of small or isolated loadings precluded substantive interpretation. For transparency, Table 6 displays the complete set of item loadings across all eight factors, but only the first three were judged meaningful and are interpreted below.

Factor 1 emerged as a broad general factor. Most TAS-20 items loaded strongly (17 of 20), including all seven DIF items (λ = 0.584 to 0.774) and all five DDF items (λ = 0.604 to 0.761). All 24 PAQ items also loaded substantially (λ = 0.420 to 0.828), as did 19 of 21 DASS-21 items (λ = 0.407 to 0.651). This factor captures substantial common variance shared between alexithymia and psychological distress and was labeled ‘General Emotional Dysfunction.’

The emergence of such a broad factor is consistent with contemporary dimensional models in which vulnerability variables share substantial variance with symptom indicators yet remain distinguishable from them (Kotov et al., 2010a, 2010b, 2017; Wright & Simms, 2015). The strong DIF and DDF loadings on General Emotional Dysfunction reflect a stable liability for emotional difficulties that occupies the same broad “emotional dysfunction” space as depressive, anxiety, and stress symptoms, without implying that alexithymia is reducible to those symptoms. Importantly, the absence of meaningful DIF/DDF cross-loadings on the more specific Psychological Distress factor (Factor 2, described below) indicates that their core variance is not simply redundant with current symptomatology. This interpretation is consistent with the correlational findings, where effect sizes fall within the range typically observed for personality vulnerability factors predicting psychopathology.

Factor 2 was identified as a ‘Psychological Distress’ factor, primarily marked by substantial and meaningful loadings from 14 of 21 DASS-21 items (λ = 0.402 to 0.573), with six additional items showing marginally meaningful loadings (λ = 0.326 to 0.381). In contrast, most TAS-20 (17 of 20) and PAQ items (18 of 24) exhibited trivial loadings. Critically, none of the seven TAS-20 DIF items cross-loaded meaningfully (all |λ| < 0.14), nor did any of the five DDF items (all |λ| < 0.20). Similarly, most PAQ DIF and DDF items showed trivial loadings. The only alexithymia items with meaningful or marginally meaningful loadings on Factor 2 were EOT items from both instruments, which loaded negatively. Three of eight PAQ G-EOT items loaded meaningfully and negatively (λ = −0.416 to −0.447), with three additional items showing marginally meaningful negative loadings (λ = −0.365 to −0.372). Three TAS-20 EOT items showed marginally meaningful negative loadings (λ = −0.335 to −0.367). The negative loading pattern replicated across both independently developed instruments, suggesting it reflects a characteristic of the EOT construct rather than a measure-specific artifact. These negative loadings do not challenge the discriminant validity of alexithymia and distress as distinct constructs, as EOT items formed a separate factor (Factor 3) distinct from both alexithymia processing deficits (DIF/DDF) and distress symptoms.

The negative loadings of EOT items on this distress factor indicate that higher externally oriented thinking was associated with lower reported distress. This loading pattern may be a measurement artifact (e.g., accurate reporting of DASS-21 symptoms requires introspection about internal feeling states, which individuals high in externally oriented thinking may be less capable of detecting, creating a methodological confound). Alternatively, this pattern of loadings may have substantive meaning (e.g., a genuine adaptive function in which reduced attention to emotions and/or a pragmatic, stimulus-bound thinking style blunts emotional distress).

Factor 3 was interpreted as an ‘Externally Oriented Thinking’ factor, characterized by meaningful or near-meaningful loadings from EOT items of both the TAS-20 (λ = 0.299–0.423) and PAQ (λ = 0.314–0.411). DIF and DDF items from both instruments showed minimal loadings on this factor. No DASS-21 items loaded meaningfully (all λ < 0.299), confirming Factor 3 as a distinct dimension of alexithymia separate from psychological distress.

The convergence of EOT items from both independently developed instruments indicates these subscales share substantial variance despite somewhat different theoretical frameworks—the TAS-20 EOT reflecting a stimulus-bound, operational mode of thinking (Taylor et al., 1997), and the PAQ EOT reflecting lack of attention to emotions (Preece et al., 2018). This supports the view that a tendency to focus on external, objective features of experience at the expense of internal emotional experiences remains a defining feature of alexithymia across measurement traditions. Factor 3 thus captures a stable cognitive-experiential orientation rather than a transient state of distress, consistent with characterizations of externally oriented thinking as a trait-like limitation in symbolic and affective capacity (Taylor et al., 2023, 2024).

The item-level factor structure provides strong evidence for discriminant validity of both the TAS-20 and the PAQ. While alexithymia and distress share broad common variance (Factor 1), the core components of both instruments—particularly DIF and DDF items—are factorially distinct from psychological distress symptomatology. The emergence of a separate EOT factor further confirms that different facets of alexithymia relate to distress in theoretically meaningful but distinct ways.

#### 3.3.2. Subscale-Level Second-Order Factor Analysis

To evaluate latent structure at the subscale level, we conducted exploratory factor analysis on the correlation matrix of TAS-20, PAQ, and DASS-21 subscales, following procedures parallel to those used for item-level analysis. This approach assessed whether alexithymia subscales formed a factor distinct from DASS-21 subscales, using a methodology similar to Preece et al. (2020, 2024). As shown in Table 3, all subscales approximated normal distributions, supporting the appropriateness of factor analysis.

The Kaiser–Meyer–Olkin (KMO) measure of sampling adequacy was 0.81, indicating that subscale correlations were suitable for factor analysis. Bartlett’s test of sphericity was significant, χ^2^(55) = 6599.39, *p* < 0.001. Inspection of communalities revealed that all subscales exceeded the recommended minimum (0.30), supporting factorability.

Parallel analysis indicated that two eigenvalues exceeded those from random data, suggesting a two-factor solution. The MAP test (Velicer, 1976) and scree plot also supported two factors. Based on these convergent findings, we extracted two factors. We used principal axis factoring with oblimin rotation to allow for correlated factors and extracted two factors, which accounted for 74.4% of the total variance. Table 7 presents factor loadings and communalities.

Mirroring the item-level analysis, both alexithymia and distress subscales loaded substantially on Factor 1, which accounted for 60.4% of variance. All three TAS-20 subscales loaded meaningfully (λ = 0.439 to 0.863), as did all five PAQ subscales (λ = 0.703 to 0.889) and all three DASS-21 subscales (λ = 0.646 to 0.650). Consistent with the item-level analysis, this factor was labeled ‘General Emotional Dysfunction.’

Factor 2 was composed of strong loadings from all three DASS-21 subscales (λ = 0.441 to 0.650). In contrast, seven of eight alexithymia subscales showed minimal or negative loadings (λ = −0.270 to 0.050). The exception was the PAQ G-EOT subscale, which showed a marginally meaningful negative loading (λ = −0.347). This factor was labeled ‘Psychological Distress’ and accounted for 14.36% of the variance.

Critically, the TAS-20 DIF subscale loaded meaningfully and strongly on the general emotional dysfunction factor (λ = 0.863), with a minimal and trivial loading on the distress factor (λ = 0.050), demonstrating clear discriminant validity. Similarly, the TAS-20 DDF subscale loaded exclusively on the general emotional dysfunction factor (λ = 0.779), with trivial loading on the distress factor (λ = −0.124). All PAQ subscales assessing difficulty identifying and describing feelings (N-DIF, P-DIF, N-DDF, P-DDF) showed the same pattern, loading substantially on Factor 1 (λ = 0.833 to 0.890) with minimal loadings on Factor 2 (λ = −0.120 to −0.213).

The subscale-level factor structure thus replicates and reinforces the item-level findings. Both alexithymia measures demonstrated structural separation from psychological distress, with alexithymia subscales and distress subscales forming distinct factors despite sharing higher-order variance. The lack of TAS-20 DIF cross-loading on the psychological distress factor in our data is particularly noteworthy, as this was Preece et al.’s primary basis for questioning the discriminant validity of the TAS-20. Our results indicate that, at least in this enriched community sample, the TAS-20 and PAQ both demonstrate adequate discriminant validity relative to psychological distress at the subscale level.

## 4. Discussion

### 4.1. Primary Findings and Theoretical Implications

This investigation evaluated the discriminant validity of the TAS-20 and PAQ relative to psychological distress using correlational and factor analytic methods at both item and subscale levels in a community sample enriched with participants having psychiatric histories. Across all analyses, including the first- and second-order factor analyses, alexithymia and distress emerged as separable yet moderately correlated constructs, providing compelling evidence that both instruments assess domains structurally distinct from general psychological distress.

Consistent with the existing body of first-order factor analytic research (see Table 1), our item-level analysis replicated the results from these studies, confirming that alexithymia and distress items are structurally separable, with no meaningful cross-loading of DIF or DDF items on the distress factors. Notwithstanding, it is at the second-order, subscale level where the question of discriminant validity has been contested, and so a more critical outcome is that our second-order factor analysis did not replicate the key finding reported by Preece et al. (2024). In their U.S. community sample (*N* = 508), the TAS-20 DIF subscale cross-loaded substantially on both the alexithymia factor and the distress factor, leading them to conclude that “substantial variance in the TAS-20 DIF subscale appears to reflect not only alexithymia, but also how distressed respondents currently were” (p. 143). In contrast, the TAS-20 DIF subscale in our study loaded strongly and exclusively on a general emotional dysfunction factor with negligible loading on the distress factor. This direct non-replication suggests that TAS-20 DIF cross-loading on distress may not be a robust or universal feature of the scale and that broad conclusions about significant discriminant validity problems may be premature.

Importantly, this finding has precedent in Preece et al.’s (2020) own multi-sample study. Across their three samples that completed the TAS-20, the pattern of DIF cross-loading was inconsistent: in Sample 1 (*n* = 300), DIF loaded 0.40 on alexithymia and −0.51 on distress (a negative loading); in Sample 2 (*n* = 128), DIF showed split loadings (0.47 on alexithymia, 0.46 on distress); and in Sample 3 (*n* = 216), DIF loaded more strongly on alexithymia (0.59) than distress (0.42).^2^ This variability across samples, including the lack of cross-loading in our data, suggests that the cross-loading phenomenon, when it occurs, may be sample-specific rather than a stable psychometric property of the TAS-20. Taken together with our findings, this variability suggests that strong conclusions based on any single study should be drawn cautiously, particularly with respect to broad statements about the relative superiority of one alexithymia measure over another or the need to reinterpret the existing TAS-20 DIF literature (see Preece et al., 2024).

Correlational analyses revealed moderate associations between both alexithymia measures and the DASS-21, with DIF and DDF subscales showing larger correlations than EOT subscales across both instruments. These correlations fall squarely within the range typically observed for personality vulnerability factors predicting psychopathology (*r* = 0.30 to 0.50; Kotov et al., 2010a, 2010b), and well below the levels suggesting construct redundancy (*r* > 0.70). These moderate correlations did not translate into structural confounding at either the item or subscale level. This pattern aligns with the theoretical view that alexithymic affect-processing deficits elevate vulnerability to distress without being reducible to distress itself (Sifneos, 1994; Taylor et al., 1997, 2024).

These cross-sectional findings also dovetail with longitudinal evidence on the TAS-20 (e.g., Luminet et al., 2001, 2007; de Timary et al., 2008; Saarijärvi et al., 2006; Thorberg et al., 2016), which indicates modest mean-level change (limited absolute stability) but consistently moderate-to-large rank-order stability (relative stability) across illness phases and treatment. Together, the present structural evidence and prior stability findings support the view that TAS-20 scores reflect an enduring, trait-like vulnerability factor that is related to, but not reducible to, concurrent distress levels.

### 4.2. Reconciling Discrepant Findings

The divergence between our results and those of Preece et al. (2020, 2024) likely reflects differences in sample composition and analytic framework. Our community sample included substantially high proportions of participants with psychiatric diagnoses (27.75%) and treatment history (39.9%), providing greater variance in both constructs and a more stringent test of discriminant validity. When both alexithymia and distress vary widely, genuine construct overlap, even if present, should be more readily detectable.

More fundamentally, our dual-level design clarifies that conclusions about construct overlap can depend critically on analytic level. From classical psychometric theory, discriminant validity is most directly assessed through relationships among manifest indicators (items) representing core construct content (Cronbach & Meehl, 1955). When TAS-20 DIF items consistently fail to cross-load with distress items, this indicates structural distinctiveness at the empirical core. Subscale-level analyses, while valuable for understanding higher-order structure, introduce shared method variance through aggregation that can create the appearance of overlap even when item-level content is distinct. Our convergent findings across both analytic levels and both the TAS-20 and PAQ demonstrate that alexithymia and distress are structurally distinct constructs regardless of analytic framework.

In the item-level analysis, EOT items from both the TAS-20 and PAQ loaded negatively on the psychological distress factor, indicating that higher externally oriented thinking was associated with lower levels of reported distress. This pattern likely reflects two possibly complementary processes. On the one hand, accurate reporting of distress symptoms on the DASS-21 requires introspection about internal feeling states (e.g., “I felt down-hearted and blue”). Individuals high in externally oriented thinking may be less capable of detecting such symptoms. On the other hand, reduced attention to emotional states and a pragmatic, stimulus-bound thinking style may reduce subjective distress. Our correlation data support this interpretation: EOT subscales showed substantially smaller associations with DASS-21 scores compared to DIF and DDF subscales. Meta-analytic research demonstrates that strategies involving reduced attention to emotional states can provide temporary relief from subjective distress, even while contributing to longer-term emotion dysregulation and psychopathology risk (Aldao et al., 2010).

This negative loading pattern, replicated across both the independently developed TAS-20 and PAQ, provides strong evidence that it reflects a genuine characteristic of externally oriented thinking. These findings also clarify an apparent inconsistency in recent work by Preece and Gross (2024), who reported that PAQ G-EOT positively predicted DASS-21 scores in regression analyses while TAS-20 EOT showed null or negative associations. By the logic of their discriminant validity argument, wherein stronger associations with distress indicate poorer discriminant validity, this pattern would suggest that PAQ EOT has poorer discriminant validity than TAS-20 EOT. This apparent contradiction may reflect genuine differences in what the two EOT subscales assess (attention to emotions vs. operatory thinking style), which would complicate direct comparisons and undercut claims of categorical measurement superiority.

### 4.3. Insights from Multi-Level Factor Analysis

Our dual-level analytical approach revealed patterns that would be completely obscured by subscale-level analysis alone. At the item level, the three facets of alexithymia as measured by both the TAS-20 and PAQ demonstrated distinct relationships with distress: DIF and DDF items showed near-zero loadings on the distress factor, whereas EOT items from both instruments loaded negatively. This differentiation has theoretical significance, suggesting that different components of alexithymia relate to psychopathology through different mechanisms. EOT may serve a short-term emotion-regulation function that temporarily reduces subjective distress awareness, while DIF and DDF represent cognitive processing deficits that operate independently of current distress levels.

This nuanced finding is invisible at the subscale level. When EOT items are aggregated with DIF and DDF items in total alexithymia scores, the negative EOT-distress association is averaged with the near-zero DIF/DDF associations, losing the theoretically informative finding that different facets relate to distress in qualitatively different ways. Moreover, the cross-instrument replication of this pattern, observed consistently in both TAS-20 and PAQ items despite their independent development and different theoretical frameworks, provides particularly compelling evidence for construct validity that can only emerge from item-level analyses.

The emergence of a general “Emotional Dysfunction” factor onto which both alexithymia and distress items loaded is consistent with contemporary dimensional models of personality and psychopathology (Kotov et al., 2017; Wright & Simms, 2015). Such higher-order factors reflect substantial shared variance in vulnerability to emotional difficulties, yet do not imply that the constructs are redundant. The critical evidence for discriminant validity lies in the fact that manifest indicators of the alexithymia construct (i.e., items or subscales), particularly DIF and DDF, did not cross-load on the specific Psychological Distress latent factor at either the item or subscale analytic level. This pattern indicates that while alexithymia and distress share a broad emotional dysfunction space, their core content remains distinct.

These considerations support the classical position that discriminant validity is most transparently evaluated at the level of manifest indicators, where construct content is represented without aggregation artifacts (Campbell & Fiske, 1959). Second-order analyses remain valuable for understanding hierarchical structure, but conclusions about construct overlap drawn exclusively from subscale-level data should be interpreted cautiously, as aggregation may obscure meaningful construct-specific patterns.

### 4.4. Strengths, Limitations, and Future Directions

This study offers several strengths relative to previous investigations of TAS-20 discriminant validity, including the use of a community sample in which a sizable proportion had a history of psychiatric problems: concurrent item- and subscale-level analyses within the same dataset, offering convergent evidence more compelling than either approach alone; a large sample size, which provided excellent power for detecting meaningful cross-loadings; and the inclusion of two theoretically distinct alexithymia measures, allowing evaluation across different operationalizations of the alexithymia construct.

Notwithstanding these strengths, there are some limitations. While our sample was enriched with individuals having psychiatric histories, participants were not recruited from treatment settings or selected based on current mental disorders. Discriminant validity patterns may differ in actively symptomatic clinical samples where both constructs are more severe. Consequently, while our sample is ‘enriched’ with psychiatric history, findings are best generalized to community adults and those with mild-to-moderate symptoms, rather than acute clinical populations. Our cross-sectional design cannot, on its own, establish the temporal stability of the alexithymia–distress distinction. Although prior longitudinal work has documented the combination of modest mean-level change and moderate-to-high rank-order stability in TAS-20 scores across treatment and illness phases, future studies that combine such designs with concurrent structural modeling would provide especially strong tests of whether alexithymia remains distinct from distress as symptoms fluctuate. The exclusive reliance on self-report measures introduces shared method variance that could obscure discriminant validity problems or, conversely, create spurious discriminant validity through method-specific variance. Multi-method approaches incorporating clinician-administered interviews (e.g., the Toronto Structured Interview for Alexithymia; Bagby et al., 2006), behavioral or performance-based tasks or physiological measures would provide more robust tests. Future research examining issues of discriminant validity as it relates to psychological distress and alexithymia should employ other methods of assessment such as clinician-rated distress or behavioral/physiological indices so as to “rule out” common-method variance explanations. As a screening measure, the DASS-21 necessarily represents only a relatively narrow operationalization of psychological distress, which is a broad construct. Therefore, the conclusion that alexithymia measures are not contaminated by distress should be interpreted specifically regarding the depression, anxiety, and stress domains assessed by the DASS-21. In a similar vein, findings may not generalize to measures with different temporal frameworks, broader symptom coverage, or different severity ranges. Finally, while our results demonstrate structural discriminant validity (i.e., alexithymia and distress form separable factors), we did not examine functional discriminant validity (i.e., whether alexithymia measures predict clinically relevant outcomes incrementally beyond variance accounted for by distress). Such incremental validity analyses would provide important complementary evidence regarding whether alexithymia captures variance that is not only statistically distinct but also functionally meaningful.

In light of these limitations, future research priorities include: hierarchical regression analyses examining whether TAS-20 and PAQ demonstrate incremental predictive validity beyond distress for clinical outcomes; multi-method alexithymia assessment; comprehensive distress assessment using measures capturing broader symptom domains and severity ranges; longitudinal designs tracking both constructs over extended periods; and finally, and perhaps most importantly, replication in clinical samples with diagnosed psychiatric disorders.

### 4.5. Clinical and Research Implications

These findings support the clinical and research utility of both the TAS-20 and the PAQ. In this sample, neither instrument showed evidence of substantial contamination by distress, and measure selection can therefore be guided primarily by theoretical orientation and study aims rather than concerns about gross discriminant validity problems. Clinicians should interpret alexithymia scores recognizing moderate correlations with distress, particularly for DIF and DDF, while understanding that such associations reflect theoretically expected vulnerability relationships rather than construct redundancy. The DIF subscales may show slightly different patterns across measures: PAQ DIF may offer assessment somewhat less colored by current distress, whereas TAS-20 DIF’s marginally stronger distress associations could reflect greater sensitivity to the complex alexithymia–psychopathology relationship without compromising structural validity. For researchers, these instruments should be viewed as complementary tools. Tests of incremental predictive validity will clarify functional discriminant validity and help determine which aspects of alexithymia most strongly predict specific outcomes beyond general distress.

## 5. Conclusions

This study provides robust evidence for the discriminant validity of the TAS-20 and PAQ. Across item- and subscale-level analyses, alexithymia and psychological distress emerged as separable constructs. Our failure to replicate recent findings of TAS-20 DIF cross-loading suggests that such overlap may be sample-specific rather than an inherent psychometric flaw. While moderate correlations between these domains exist, they reflect theoretically expected patterns for a personality vulnerability factor rather than construct redundancy. These results affirm the continued utility of the TAS-20 in clinical and research settings and underscore the necessity of using multi-level factor analysis to distinguish genuine construct confounding from measurement artifacts.

## Figures and Tables

**Table 1:** First-order (item-level) factor analytic studies of the TAS-20 and psychological distress measures.

Study	N (Sample Description)	Alexithymia Measures	Distress Measure(s)	Extraction/ Rotation	# Factors/% Var Expl.	Key Findings
Parker et al. (1991)	406 undergraduates (Canada)	TAS-26	BDI	PCA/Varimax	4/27.3%	BDI items distinct; TAS items form separate factors with no cross-loadings on the BDI factor.
Parker et al. (1991)	164 psychiatric outpatients	TAS-26	BDI	PCA/Varimax	4/34.9%	BDI items distinct; TAS factors replicate student sample; one BDI item cross-loads on EOT.
Hintikka et al. (2001)	1888 Finnish community (25–64 yrs)	TAS-20	BDI	PCA/Varimax	6/48.6%	TAS and BDI factors distinct; minor overlap (2 TAS-20 items); weak correlations.
Marchesi et al. (2000)—a combined sample	113 patients with depressive or anxiety disorders and 113 patient controls (Italy)	TAS-20	HADS	PCA/Varimax	5/39.7%	TAS-20 and HADS items largely loaded on distinct factors; with 4 (DIF items) cross loadings.
Müller et al. (2003)—2 separate samples	199 inpatients + 174 controls (Germany)	TAS-20	SDS	PCA/Oblimin; CFA	4/41.3%	Distinct TAS and SDS factors; 1 TAS item cross-load; *r* ≈ 0.37–0.55.
Bach et al. (1996)—2 separate samples	379 non-clinical + 125 psychosomatic (Austria/Germany)	TAS-20	SOMS	PCA/Varimax	4/39–43%	TAS and SOMS separate; no overlap; *r* = 0.14–0.19; supports discriminant validity.

*Note*. TAS-20 = 20-item Toronto Alexithymia Scale; TAS-26 = 26-item precursor; BDI = Beck Depression Inventory; HADS = Hospital Anxiety and Depression Scale; SDS = Self-Rating Depression Scale; SOMS = Screening List for Somatization Symptoms; DIF = Difficulty Identifying Feelings; EOT = Externally Oriented Thinking; PCA = principal components analysis; CFA = confirmatory factor analysis. “# Factors/% Var Expl.” = number of extracted factors and total variance explained. All analyses used orthogonal (varimax) or oblique (oblimin) rotations as reported.

**Table 2:** Second-order (subscale-level) factor analytic studies of the TAS-20 and psychological distress measures.

Study	N (Sample Description)	Alexithymia Measures	Psychopathology/ Distress Measures	Extraction/ Rotation	# Factors/% Var Expl.	Key Findings
Badura (2003)	155 male U.S. combat veterans (WWII–Gulf War; VA Iowa City)	TAS-R (23-item Revised TAS)	MPTSD, Combat Exposure Scale	PCA/Varimax	1/68%	Single factor (PTSD) with all TAS-R and PTSD subscales loading 0.75–0.88; no distinct alexithymia factor; alexithymia overlaps with emotional numbing.
Leising et al. (2009)	63 adults (34 psychiatric inpatients; 29 controls; Germany)	TAS-20 (German)	SCL-90-R (9 subscales), IIP-64 (8 subscales)	PCA/Varimax	1/60.5%	One general distress/alexithymia factor TAS-20 overlaps with psychopathology and interpersonal distress; limited discriminant validity.
Preece et al. (2020, Sample 1)	300 Australian community adults	TAS-20, BVAQ	DASS-21	PAF/Oblimin	2/69.9%	Two correlated higher-order factors (alexithymia vs. general distress); *r* = −0.40 TAS-20 DIF subscale cross-loads negatively on general distress (−0.51)
Preece et al. (2020, Sample 2)	128 community + students (Australia)	TAS-20, BVAQ	DASS-21	PAF/Oblimin	2/67.9%	Two correlated higher-order factors (alexithymia vs. general distress); *r* = 0.26. TAS-20 DIF subscale cross-loads positively on general distress (0.46)
Preece et al. (2020, Sample 3)	216 Australian community	TAS-20	DASS-21	PAF/Oblimin	2/74.4%	Two correlated higher-order factors (alexithymia vs. general distress); *r* = 0.33. DIF subscale cross-loads on general distress (0.42)
Preece et al. (2020, Sample 4)	148 community + students (Australia)	PAQ	DASS-21	PAF/Oblimin	2/76%	Two correlated higher-order factors (alexithymia vs. general distress); *r* = 0.42
Preece et al. (2020, Sample 5)	103 Australian community adults	PAQ	DASS-21	PAF/Oblimin	2/77.5%	Two correlated higher-order factors (alexithymia vs. general distress); *r* = 0.40
Preece et al. (2024)	508 U.S. community adults (49.6% female)	TAS-20, PAQ	DASS-21	PAF/Oblimin	2/64.9%	Replicated two-factor structure (alexithymia vs. general distress); TAS-20 and PAQ subscales load on alexithymia; DASS-21 on distress; *r* = 0.45; the TAS-20 DIF subscale also cross-loads on the distress factor (0.50).

*Note.* TAS-R = Revised Toronto Alexithymia Scale; TAS-20 = 20-item Toronto Alexithymia Scale; BVAQ = Bermond–Vorst Alexithymia Questionnaire; PAQ = Perth Alexithymia Questionnaire; MPTSD = Mississippi Scale for Combat-Related PTSD; SCL-90-R = Symptom Checklist–90–Revised; IIP-64 = Inventory of Interpersonal Problems–64; DASS-21 = Depression Anxiety Stress Scales–21; PCA = principal components analysis; PAF = principal axis factoring; “# Factors/% Var Expl.” = number of extracted factors and total variance explained. All analyses used orthogonal (varimax) or oblique (oblimin) rotations as reported.

**Table 3:** Descriptive statistics for the TAS-20, PAQ and DASS-21.

Subscale	*M*	*SD*	Skewness	Kurtosis
TAS-20				
DIF	14.40	6.69	0.79	−0.34
DDF	12.42	5.03	0.41	−0.68
EOT	18.60	4.68	0.11	−0.45
Total	45.41	13.58	0.48	−0.37
PAQ				
P-DIF	9.60	5.46	1.09	0.55
P-DDF	11.06	5.96	0.71	−0.38
N-DIF	16.65	6.05	0.87	−0.11
N-DDF	12.40	6.70	0.56	−0.78
G-EOT	23.53	1.80	0.68	−0.24
Total	67.17	3.67	0.70	−0.19
DASS-21				
DEP	12.46	5.71	0.97	−0.04
ANX	11.58	4.17	1.52	1.98
STR	12.50	4.93	0.87	0.15
Total	35.55	13.26	1.05	0.55

*Note*. *N* = 681. *M* = mean; *SD* = standard deviation; TAS-20 = Toronto Alexithymia Scale; DIF = Difficulty Identifying Feelings; DDF = Difficulty Describing Feelings; EOT = Externally Oriented Thinking; PAQ = Perth Alexithymia Questionnaire; P-DIF = Positive-Difficulty Identifying Feelings; P-DDF = Positive-Difficulty Describing Feelings; N-DIF = Negative-Difficulty Identifying Feelings; N-DDF = Negative-Difficulty Describing Feelings; DASS-21 = Depression Anxiety Stress Scales.

**Table 4:** Internal reliability estimates for TAS-20, PAQ, and DASS-21.

Subscale	ω^t^	α	AIC	k (Items)
TAS-20				
DIF	0.91	0.91	0.59	7
DDF	0.84	0.84	0.51	5
EOT	0.62	0.64	0.18	8
Total	0.92	0.90	0.30	20
PAQ				
P-DIF	0.92	0.92	0.74	4
N-DIF	0.92	0.92	0.73	4
P-DDF	0.92	0.92	0.73	4
N-DDF	0.93	0.93	0.76	4
G-EOT	0.92	0.92	0.59	8
Total	0.97	0.97	0.56	24
DASS-21				
DEP	0.93	0.93	0.66	7
ANX	0.85	0.85	0.46	7
STR	0.90	0.90	0.55	7
Total	0.95	0.95	0.47	21

*Note. N* = 681. ω^t^ = Coefficient omega, α = Coefficient alpha, AIC = average Inter-item correlation, k = number of items; DIF = Difficulty Identifying Feelings; DDF = Difficulty Describing Feelings; EOT = Externally Oriented Thinking; TAS-20 = Toronto Alexithymia Scale; PAQ = Perth Alexithymia Questionnaire; P-DIF = Positive-Difficulty Identifying Feelings; P-DDF = Positive-Difficulty Describing Feelings; N-DIF = Negative-Difficulty Identifying Feelings; N-DDF = Negative-Difficulty Describing Feelings; DASS-21 = Depression Anxiety Stress Scales.

**Table 5:** Correlations between the TAS-20, PAQ, and DASS-21 subscales.

	DASS-21 Subscales			
Scale	Depression	Anxiety	Stress	Total
TAS-20				
DIF	0.57	0.55	0.58	0.63
DDF	0.51	0.44	0.49	0.53
EOT	0.21	0.12	0.15	0.18
Total	0.54	0.47	0.52	0.57
PAQ				
N-DIF	0.49	0.46	0.51	0.54
P-DIF	0.48	0.39	0.45	0.50
N-DDF	0.48	0.40	0.46	0.50
P-DDF	0.47	0.34	0.45	0.47
G-EOT	0.34	0.20	0.25	0.30
Total	0.50	0.37	0.46	0.50

*Note. N* = 681. PAQ = Perth Alexithymia Questionnaire; P-DIF = Positive-Difficulty Identifying Feelings; P-DDF = Positive-Difficulty Describing Feelings; N-DIF = Negative-Difficulty Identifying Feelings; N-DDF = Negative-Difficulty Describing Feelings; TAS-20 = Toronto Alexithymia Scale; DIF = Difficulty Identifying Feelings; DDF = Difficulty Describing Feelings; EOT = Externally Oriented Thinking; DASS-21 = Depression, Anxiety, and Stress Scales. All correlations were significant at *p* < 0.001.

**Table 6:** Results for first-order (item-level) factor analysis—eight factor solution.

	Standardized Factor Loadings								Communalities	
Scale/Items	F1	F2	F3	F4	F5	F6	F7	F8	Initial	Extraction
TAS-20										
Q1 (DIF)	**0.775**	−0.007	−0.231	0.096	0.197	−0.042	−0.084	0.051	0.760	0.714
Q2 (DDF)	**0.737**	−0.097	−0.226	0.054	0.211	−0.255	0.095	0.010	0.755	0.725
Q3 (DIF)	**0.584**	0.067	−0.101	0.107	0.134	0.240	−0.012	0.087	0.554	0.45
Q4 (DDF)	**0.629**	−0.188	−0.087	0.011	0.230	−0.220	0.114	0.031	0.597	0.553
Q5 (EOT)	−0.093	−0.058	0.053	−0.026	0.202	0.089	**0.434**	0.100	0.261	0.262
Q6 (DIF)	**0.744**	−0.085	−0.223	0.084	0.117	0.051	−0.126	0.098	0.707	0.66
Q7 (DIF)	**0.632**	0.136	−0.148	0.184	0.110	0.263	−0.019	0.180	0.642	0.588
Q8 (EOT)	***0.323***	−0.229	0.160	0.012	−0.015	0.090	0.292	0.080	0.340	0.283
Q9 (DIF)	**0.762**	−0.034	−0.289	0.058	0.117	−0.009	−0.071	0.084	0.713	0.695
Q10 (EOT)	0.297	***−0.364***	0.299	0.067	0.108	−0.018	0.199	0.145	0.419	0.387
Q11 (DDF)	**0.761**	−0.122	−0.149	0.008	0.125	−0.122	0.018	−0.015	0.679	0.648
Q12 (DDF)	**0.604**	−0.096	−0.066	0.123	−0.024	−0.056	−0.012	−0.142	0.487	0.418
Q13 (DIF)	**0.774**	0.009	−0.152	0.047	0.183	0.043	−0.046	0.122	0.719	0.676
Q14 (DIF)	**0.633**	0.057	−0.165	0.148	−0.021	0.060	−0.077	0.244	0.525	0.522
Q15 (EOT)	***0.312***	***−0.335***	***0.373***	0.191	−0.169	−0.081	−0.047	0.090	0.490	0.431
Q16 (EOT)	0.173	−0.076	0.117	0.137	−0.097	−0.002	0.033	0.201	0.257	0.119
Q17 (DDF)	**0.615**	−0.119	0.225	0.032	−0.022	−0.234	−0.058	−0.112	0.573	0.515
Q18 (EOT)	0.292	−0.036	0.158	−0.077	0.194	−0.015	0.201	0.082	0.267	0.202
Q19 (EOT)	***0.339***	***−0.367***	**0.423**	−0.044	0.174	−0.041	***0.322***	0.172	0.528	0.596
Q20 (EOT)	0.285	−0.053	0.094	0.184	−0.109	0.116	0.022	0.111	0.283	0.165
PAQ										
Q1 (N-DDF)	**0.776**	−0.175	−0.259	0.056	0.087	−0.177	0.022	−0.068	0.770	0.747
Q2 (N-DIF)	**0.756**	−0.120	−0.245	0.082	0.075	0.050	−0.075	0.038	0.740	0.667
Q3 (G-EOT)	**0.689**	−0.290	0.219	0.130	0.090	−0.053	−0.086	−0.141	0.721	0.662
Q4 (P-DDF)	**0.735**	−0.187	−0.157	−0.191	−0.193	−0.001	0.073	−0.029	0.744	0.681
Q5 (P-DIF)	**0.717**	−0.139	−0.219	−0.253	−0.123	0.114	0.071	0.025	0.711	0.679
Q6 (G-EOT)	**0.465**	**−0.416**	***0.355***	0.104	−0.076	0.098	−0.083	−0.013	0.595	0.549
Q7 (N-DDF)	**0.744**	−0.246	0.062	0.004	0.037	−0.173	0.041	−0.123	0.726	0.666
Q8 (N-DIF)	**0.828**	−0.159	−0.176	0.064	0.067	0.065	−0.094	0.009	0.792	0.763
Q9 (G-EOT)	**0.609**	**−0.447**	0.278	0.103	0.023	0.033	−0.136	−0.092	0.721	0.688
Q10 (P-DDF)	**0.672**	−0.281	0.001	−0.279	−0.194	−0.086	0.061	−0.069	0.695	0.662
Q11 (P-DIF)	**0.763**	−0.188	−0.136	−0.286	−0.203	0.174	0.051	−0.026	0.806	0.792
Q12 (G-EOT)	**0.700**	−0.274	***0.314***	0.088	−0.062	0.074	−0.068	−0.076	0.712	0.691
Q13 (N-DDF)	**0.828**	−0.197	−0.135	0.067	0.033	−0.157	−0.017	−0.109	0.827	0.786
Q14 (N-DIF)	**0.801**	−0.107	−0.276	0.044	0.043	0.107	−0.095	0.061	0.792	0.758
Q15 (G-EOT)	**0.523**	***−0.367***	***0.327***	0.162	−0.110	0.052	−0.158	0.048	0.600	0.583
Q16 (P-DDF)	**0.816**	−0.228	−0.097	−0.252	−0.176	−0.032	0.078	−0.059	0.836	0.832
Q17 (P-DIF)	**0.777**	−0.191	−0.205	−0.202	−0.140	0.179	0.048	0.032	0.812	0.778
Q18 (G-EOT)	**0.557**	**−0.431**	**0.411**	0.091	−0.066	0.069	−0.070	−0.001	0.683	0.688
Q19 (N-DDF)	**0.828**	−0.174	−0.137	0.052	0.075	−0.203	0.037	−0.118	0.849	0.800
Q20 (N-DIF)	**0.774**	−0.137	−0.201	0.100	−0.021	0.053	−0.018	0.001	0.752	0.671
Q21 (G-EOT)	**0.420**	***−0.372***	0.262	0.052	−0.042	0.115	−0.053	0.050	0.467	0.406
Q22 (P-DDF)	**0.771**	−0.199	−0.109	−0.193	−0.201	−0.034	0.142	−0.098	0.774	0.754
Q23 (P-DIF)	**0.726**	−0.127	−0.197	−0.182	−0.168	0.233	0.042	−0.017	0.722	0.700
Q24 (G-EOT)	**0.686**	***−0.365***	0.216	0.027	−0.013	0.074	−0.026	−0.109	0.690	0.670
DASS−21										
Q1 (STR)	**0.579**	***0.352***	0.004	0.065	−0.178	−0.160	0.025	0.107	0.660	0.532
Q2 (ANX)	0.316	0.256	0.025	0.050	0.030	0.116	0.087	−0.161	0.297	0.216
Q3 (DEP)	**0.651**	***0.370***	0.172	−0.219	0.051	0.030	−0.047	0.057	0.709	0.647
Q4 (ANX)	**0.475**	***0.381***	0.075	0.143	0.021	0.177	0.115	−0.149	0.536	0.464
Q5 (DEP)	**0.545**	**0.410**	0.173	−0.099	0.097	−0.093	−0.089	−0.012	0.586	0.531
Q6 (STR)	**0.506**	**0.458**	−0.011	0.063	−0.187	−0.125	0.034	0.146	0.581	0.544
Q7 (ANX)	**0.407**	***0.326***	0.068	0.225	−0.019	0.105	0.176	−0.214	0.422	0.415
Q8 (STR)	**0.517**	**0.520**	0.068	0.151	−0.152	0.019	0.129	−0.047	0.614	0.607
Q9 (ANX)	**0.478**	**0.526**	0.048	0.127	−0.021	0.006	0.115	−0.184	0.619	0.572
Q10 (DEP)	**0.585**	**0.417**	0.289	***−0.334***	0.164	0.043	−0.108	0.018	0.730	0.752
Q11 (STR)	**0.530**	**0.471**	0.054	0.090	−0.150	−0.156	0.038	0.166	0.601	0.590
Q12 (STR)	**0.580**	**0.469**	0.103	0.029	−0.177	**−0.210**	0.024	0.051	0.716	0.647
Q13 (DEP)	**0.560**	**0.531**	0.205	−0.197	0.070	−0.036	−0.088	0.109	0.707	0.702
Q14 (STR)	**0.462**	**0.424**	0.043	0.075	−0.261	−0.088	−0.040	0.129	0.519	0.495
Q15 (ANX)	**0.492**	**0.573**	0.018	0.234	−0.025	0.101	0.058	−0.129	0.665	0.657
Q16 (DEP)	**0.639**	***0.369***	0.277	−0.274	0.108	0.008	−0.069	0.083	0.735	0.720
Q17 (DEP)	**0.599**	**0.422**	0.247	−0.257	0.224	0.025	−0.085	−0.062	0.742	0.726
Q18 (STR)	**0.480**	**0.402**	0.043	0.092	−0.221	−0.121	0.028	0.133	0.507	0.485
Q19 (ANX)	***0.371***	**0.461**	0.029	0.186	0.096	0.154	0.103	−0.146	0.490	0.451
Q20 (ANX)	**0.492**	**0.516**	−0.004	0.108	−0.023	0.100	0.019	−0.106	0.617	0.542
Q21 (DEP)	**0.559**	***0.372***	0.299	−0.240	0.184	0.067	−0.132	−0.043	0.688	0.656
Eigenvalue	24.70	6.64	2.97	1.81	1.59	1.40	1.35	1.25		
% Variance Explained	37.99	10.22	4.57	2.78	2.45	2.15	2.08	1.92		
Cumulative % Variance	37.99	48.21	52.78	55.56	58.01	60.16	62.23	64.16		

*Note*: *N* = 681. TAS-20 = Toronto Alexithymia Scale—20-item; PAQ = Perth Alexithymia Questionnaire; DASS-21 = Depression, Anxiety, and Stress Scale; bolded = salient factor loading (i.e., ≥0.40); bold and italics = marginally salient loading (i.e., ≥0.30 and <0.40); non-meaningful loadings are <0.30, but >0.20; trivial loadings are ≤0.20.

**Table 7:** Results for second-order (subscale-level) factor analysis.

	Standardized Factor Loadings		Communalities	
Scales	General Emotional Dysfunction	Psychological Distress	Initial	Extraction
TAS-20				
DIF	**0.863**	0.050	0.780	0.747
DDF	**0.862**	−0.124	0.770	0.759
EOT	**0.439**	−0.270	0.358	0.266
PAQ				
N-DIF	**0.889**	−0.120	0.857	0.805
P-DIF	**0.833**	−0.146	0.833	0.716
N-DDF	**0.890**	−0.198	0.852	0.831
P-DDF	**0.847**	−0.213	0.841	0.762
G-EOT	**0.703**	***−0.347***	0.616	0.614
DASS-21				
STR	**0.646**	**0.575**	0.638	0.748
ANX	**0.568**	**0.620**	0.591	0.707
DEP	**0.650**	**0.441**	0.583	0.617
Eigenvalue	6.60	1.58		
% Variance Explained	60.04	14.36		
Cumulative % Variance	60.04	74.40		

*Note*: *N* = 681; PAQ = Perth Alexithymia Questionnaire, P-DIF = Positive-Difficulty Identifying Feelings, P-DDF = Positive-Difficulty Describing Feelings, N-DIF = Negative-Difficulty Identifying Feelings, N-DDF = Negative-Difficulty Describing Feelings; TAS-20 = Toronto Alexithymia Scale; DIF = Difficulty Identifying Feelings; DDF = Difficulty Describing Feelings; EOT = Externally Oriented Thinking; DASS-21 = Depression Anxiety Stress Scales. Bolded = salient factor loading (i.e., ≥0.40); bold and italics = marginally salient loading (i.e., ≥0.30 and <0.40); non-meaningful loadings are <0.30 but >0.20; trivial loadings are ≤0.20.

## Data Availability

The data that support the findings of this study are available from the corresponding author upon reasonable request.

## Notes

1 The limited capacity for imagination of the alexithymia construct is captured, in part, by the EOT factor subscale, as it is thought that those with stimulus-bound thinking, by default, have limited imagination, and also by empirical studies demonstrating medium effect-size associations between the TAS-20 EOT subscale and measures of reduced imaginal capacity. However, as noted by Taylor et al. (2024), the restricted imaginal capacity facet of the construct is difficult to assess directly with self-report scales; it is best assessed with the Toronto Structured Interview for Alexithymia (Bagby et al., 2006) or with performance-based tests.

2 Preece et al. (2020) indicate that in Sample 1, the EFA extracted the general distress factor as a “lack of general distress” factor.
